# Questions regarding the reduction of the incidence of stage 4 breast cancer in Europe—authors’ reply

**DOI:** 10.1016/j.lanepe.2026.101661

**Published:** 2026-03-21

**Authors:** Rafael Cardoso, Idris Ola, Hermann Brenner

**Affiliations:** a Division of Clinical Epidemiology of Early Cancer Detection, German Cancer Research Center (DKFZ), Heidelberg, Germany; b Medical Faculty Heidelberg, University of Heidelberg, 69120, Heidelberg, Germany; c Cancer Prevention Graduate School, German Cancer Research Center (DKFZ), Heidelberg, Germany

We thank Philippe Autier for the interest in our paper.^1^ Firstly, we would like to clarify that we conducted multiple imputation on each registry dataset individually to address the varying proportion of cases with unknown stage over time.^2^ Contrary to the selective citation by Philippe Autier, we reported the full range of average annual percent changes (AAPCs) in stage IV incidence after screening introduction, from stable rates in the Netherlands (−0.40 (95% CI −2.17 to 1.40)) down to −6.16 (95% CI −8.28 to −4.00) in South Portugal; we refer to the Abstract and Table 4 of the manuscript.^2^ Importantly, we refer to and discuss the large heterogeneity of trends across European countries multiple times throughout the manuscript, including in the concluding paragraph of the Discussion.

Lastly, we fully agree with the comments regarding changes in cancer registration over time, which we discuss at length in the Discussion: “missing or unknown staging information (whose proportion varied over time across countries) was imputed to minimise biases in the interpretation of trends in stage-specific incidence. Nevertheless, stage-specific incidence trends should be interpreted with caution, especially in countries with a high proportion of unknown stage. Third, a likely stage migration (from stage II to III) might have occurred over time, particularly around 2003, due to reclassification of tumours with more than three positive lymph nodes as stage III instead of stage II. This was noticeable for the Netherlands, Belgium (Flanders), and Germany among 0–49-year-olds.”^2^

In summary, while we appreciate the comments by Philippe Autier, we continue to think that we followed the analytical approach that best serves the data at hand, while explicitly acknowledging its important merits and limitations.

## Contributors

RC, IO, and HB contributed to the drafting of this response.

## Declaration of interests

RC was employed by The Lancet Regional Health—Europe as a senior editor from 1st April 2024 to 30th September 2025. He had no involvement in the peer review for this Correspondence, or the original Article, where he is an author. IO and HB have no conflicts of interest to declare.
